# Personalized SO_2_ Prodrug for pH-Triggered Gas Enhancement in Anti-Tumor Radio-Immunotherapy

**DOI:** 10.3390/pharmaceutics16060833

**Published:** 2024-06-19

**Authors:** Zhiran Chen, Xiaoxiang Zhou, Bo Wu, Han Tang, Wei Wei, Daoming Zhu, Yi Ding, Longyun Chen

**Affiliations:** 1 The Yancheng School of Clinical Medicine of Nanjing Medical University, Yancheng Third People’s Hospital, Yancheng 224051, China; kk18751426705@163.com (Z.C.); xiaoxiang.zhou@outlook.com (X.Z.); wubo60912@163.com (B.W.); 2 Key Laboratory of Artificial Micro- and Nano-Structures of Ministry of Education, School of Physics and Technology, Wuhan University, Wuhan 430072, China; th1998@whu.edu.cn; 3 Department of Radiation Oncology, Hubei Cancer Hospital, TongJi Medical College, Huazhong University of Science and Technology, Wuhan 430030, China; ww_hbch@163.com; 4 Department of General Surgery, Guangdong Provincial Key Laboratory of Precision Medicine for Gastrointestinal Tumor, Nanfang Hospital, Southern Medical University, Guangzhou 510515, China; zhudaoming666@smu.edu.cn

**Keywords:** benzothiazole sulfinate, tumor microenvironment, gas therapy, immunotherapy, radiotherapy

## Abstract

The inhibition of the immune response in the tumor microenvironment by therapy regimens can impede the eradication of tumors, potentially resulting in tumor metastasis. As a non-invasive therapeutic method, radiotherapy is utilized for tumor ablation. In this study, we aimed to improve the therapeutic impact of radiotherapy and trigger an immune response by formulating a benzothiazole sulfinate (BTS)-loaded fusion liposome (BFL) nanoplatform, which was then combined with radiotherapy for anti-cancer treatment. The platelet cell membrane, equipped with distinctive surface receptors, enables BFL to effectively target tumors while evading the immune system and adhering to tumor cells. This facilitates BFL’s engulfment by cancer cells, subsequently releasing BTS within them. Following the release, the BTS produces sulfur dioxide (SO_2_) for gas therapy, initiating the oxidation of intracellular glutathione (GSH). This process demonstrates efficacy in repairing damage post-radiotherapy, thereby achieving effective radiosensitization. It was revealed that an immune response was triggered following the enhanced radiosensitization facilitated by BFL. This approach facilitated the maturation of dendritic cell (DC) within lymph nodes, leading to an increase in the proportion of T cells in distant tumors. This resulted in significant eradication of primary tumors and inhibition of growth in distant tumors. In summary, the integration of personalized BFL with radiotherapy shows potential in enhancing both tumor immune response and the elimination of tumors, including metastasis.

## 1. Introduction

Gas therapy, emerging as a novel treatment strategy, is currently garnering significant attention. By leveraging the physical and chemical properties of nanoplatforms, which generate therapeutic gases like hydrogen (H_2_) [1,2], sulfur dioxide (SO_2_) [3,4], hydrogen sulfide (H_2_S) [5,6], and nitric oxide (NO) [7,8,9] through prodrug activation and donation, gas therapy demonstrates promise in modulating the physiological and chemical conditions to treat various diseases, such as inflammation, cardiac ischemia-reperfusion, and cancer [10,11,12]. In tumor therapy, nanoplatforms that generate SO_2_ within tumors can effectively address tumor heterogeneity, leading to tumor eradication. Previous research developed a nanocomplex for SO_2_ release to diminish cancer stem cells in vivo, indicating its potential in modulating the issues of tumor heterogeneity [3]. A recently developed copper-loaded SO_2_ prodrug has been found to exhibit the capacity to reduce levels of reducible glutathione (GSH) and enhance the production of reactive oxygen species (ROS), thereby showing promise for anticancer applications [4]. Another bimetallic gold–silver-based nanorattle was developed as carriers for SO_2_ prodrugs, enabling controlled release of gas to increase SO_2_ levels for enhanced generation of ROS [13]. At the same time, layered MgAl nanosheets containing inserted sulfite can induce both starvation therapy and gas therapy [14]. Nevertheless, it is crucial to consider the potential toxicity of nanoplatforms that generate SO_2_ to both the body and adjacent normal tissues surrounding tumors.

Consideration of the targeting efficiency is essential before applying a nanoplatform for tumor treatment, as this approach can help reduce overall systemic toxicity while enhancing therapeutic efficacy [15,16,17]. In recent times, personalized tumor therapy methods tailored to an individual patient’s physical conditions are demonstrating promising potential [18,19]. Among these approaches, the modification of nanoplatforms using homologous cell membranes is gaining attention [20,21,22]. Derived from natural cells, the extracted membrane retains complex functionalities inherited from cells, thereby imparting specific characteristics to the mimicked nanoplatform [23,24,25]. For instance, membranes extracted from red blood cell were endowed with properties including long-circulating time, biocompatibility, and biodegradability [26,27]. Platelet membrane contributes to enhancing selectively targeting ability and higher cellular uptake [28,29]. Nanoparticles cloaked with cancer cell membranes inherited immune escape and tumor-homing features from cancer cell [3,19]. The fabrication methods for membrane-coated nanoplatforms include physical extrusion, sonification, and electroporation [30,31,32]. In contrast to source cells like red blood cells, platelet cells can not only evade immune system clearance by “marker of self” CD47, but also exhibit both passive and active tumor-targeting abilities due to their unique surface receptors such as CD44 or p-selectin glycoprotein ligand-1 [33,34,35]. A prior study conducted by Dong et al. introduced a nanoparticle covered with a platelet cell membrane for photothermal therapy targeting hepatocellular carcinoma [20]. A different nanococktail featuring platelet camouflage was utilized to alleviate drug resistance through dual targeting of both cancer cells and the vasculature [36]. Chen reported a magnetic metal–organic framework nanoplatform coated with platelet cell membranes to enhance immunotherapy for tumor eradication [37]. Nonetheless, the limited penetration depth of nanodrugs suggests that the therapeutic agents may not effectively reach tumor cells located deep within tissues, far from vasculatures. Consequently, incomplete tumor ablation may lead to the occurrence of tumor metastasis following treatment.

**Scheme 1:**
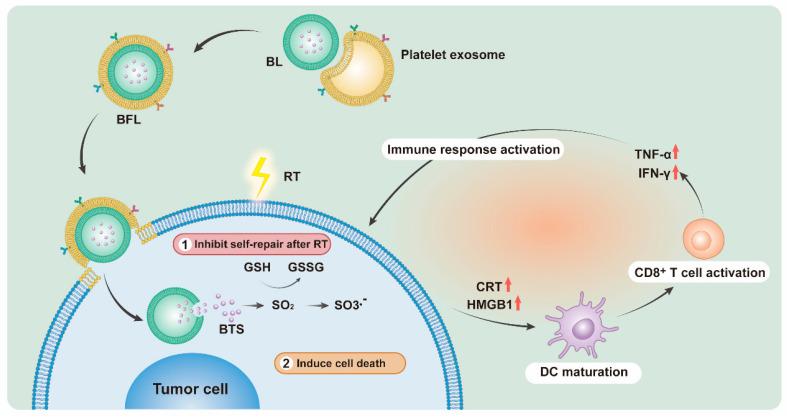
The mechanism of BFL enhanced RT for immune response activation.

Ionizing radiation which delivers substantial energy beams, such as electron, proton, and photon, to a target is extensively employed for cancer treatment due to its effective penetration depth, yielding satisfactory therapeutic outcomes to alleviate tumors. However, procedural errors including the positioning error and respiratory movement during radiotherapy (RT) can contribute to the occurrence of metastasis due to incomplete elimination of tumors [38]. Hence, we developed a novel benzothiazole sulfinate (BTS)-loaded fusion liposome (BFL) nanoplatform composed of platelet cell membranes and lipid fusion camouflaged nanoparticles. This platform is designed for the delivery of BTS, a prodrug of SO_2_, to facilitate combination treatment with RT (Scheme 1). When comparing BTS with lipid coating alone (BL), the fusion of lipid and platelet cell membranes significantly enhances cellular uptake in vitro and tumor targeting in vivo of BFL. Once released from the tumor vasculature, BFL is internalized by cells and releases BTS intracellularly. In the mildly acidic tumor microenvironment, BTS liberates ample SO_2_, which, on one hand, diminishes the GSH levels within tumor cells and, on the other hand, transforms into ·SO_3_^−^ to induce cell death. As is widely understood, ionizing irradiation leads to the generation of ROS, causing DNA damage and ultimately tumor ablation. Nonetheless, the reducible GSH can repair this damage by reducing the amount of ROS. However, due to the oxidizing effect of SO_2_ on GSH, the ROS generated from ionizing irradiation can be preserved, thereby maintaining the overall treatment effect. Surprisingly, our approach demonstrated an immunogenic cell death (ICD) effect, which enhanced dendritic cell (DC) maturation in lymph nodes and consequently increased T cell infiltration in distant tumors. These findings indicate the potential of our strategy for both tumor eradication and inhibition of metastasis.

## 2. Materials and Methods

### 2.1. Materials

Cholesterol (CHOL) and 1,2-dipalmitoyl-sn-glycero-3-phosphocholine (DPPC) were purchased from A.V.T (Shanghai, China). The high mobility group protein box 1 (HMGB1) enzyme-linked immunosorbent assay (ELISA) kit was purchased from Beijing Solarbio Science & Technology Co., Ltd. (Beijing, China). All of the aqueous solutions were prepared using purified deionized (DI) water purified with a purification system (Direct-Q3, Millipore, Burlington, MA, USA). The other solvents used in this work were purchased from Sinopharm Chemical Reagent (China, Shanghai) and Aladdin-Reagent (China, Shanghai).

### 2.2. Preparation and Characterization of BTS Loaded Fusion Liposomes (BFL)

The platelet membranes (PM) were prepared according to previous work [39]. Platelets from 10 mL of mice whole blood were isolated and centrifuged at 100× *g* for 20 min. Then the platelet suspensions were first frozen at −80 °C, thawed at room temperature, and pelleted by centrifugation at 4000× *g* for 3 min. After three repeated washes with PBS mixed with protease inhibitor tablets, the membranes were suspended in water, sonicated in a capped glass vial for 5 min using a bath sonicator, and then extruded sequentially through 400 and 200 nm polycarbonate porous membranes on a mini extruder (Avanti Polar Lipids, Alabaster, AL, USA) to form PM.

Then, 27 mg DPPC and 2.8 mg CHOL were dissolved in chloroform, and then evaporated at 55 °C for 80 min to form a thin film in a rotary evaporator. Then the film was hydrated with PBS solution containing PM (3 mg) and BTS (3 mg) at 37 °C for 5 min and subjected to ultrasound followed by repeated extrusion through 100-nm polycarbonate pores. The resultant BFL particles were dialyzed overnight in a dialysis bag (MWCO 300 kD) to remove unencapsulated BTS. BTS loading capacity was calculated by UV–vis spectra at the UV–vis spectrophotometry Lambda 35 (Perkin-Elmer, Waltham, MA, USA). Loading capacity = M_drug_/M_BFL_. where M refers to the mass. The BTS-loaded liposomes (BL) were prepared in the same way, except for replacing the PM with PBS. The zeta potential and diameter of nanoparticles were measured by dynamic light scattering (DLS, Nano-Zen 3600, Malvern Instruments, UK, Worcestershire). The morphological structures of different formulations and the elemental mapping images were captured by transmission electron microscopy (TEM, JEM-2100F, JEOL, Japan, Tokyo).

### 2.3. SO_2_ Release

7-Diethylaminocoumarin-3-aldehyde (DEACA) was applied for quantification analysis of bisulfite. In the presence of bisulfite, DEACA changed to blue fluorescent selectively. An amount of 5 μM of DEACA solution was added into the BFL (containing 0.05 mg BTS) solutions under different pH values. Next, a fluorescence spectrophotometer was utilized to measure the blue fluorescence intensity.

### 2.4. Tumor Cell Targeting Assessment

4T1 cells were seeded in 24-well plates for tumor cell targeting assessment. After 12 h, the DiO-labeled BFL and BL containing 50 μg/mL BTS were added for co-incubation. Next, 4T1 cells were then fixed and cell nuclei was stained with DAPI. A confocal laser scanning microscope was applied for fluorescence observation (CLSM; IX81, Olympus, Japan, Tokyo).

### 2.5. ROS, DNA Damage, and ICD Detection

4T1 cells were seeded in 24-well plates and divided into the following five different groups: (1) PBS; (2) RT (4 Gy); (3) BFL; (4) BL + RT; (5) BFL + RT, containing 50 μg/mL BTS. Two hours after adding BL and BFL, RT was delivered. Next, the ROS fluorescent dye DCFH-DA (10 μM) or anti-γH_2_AX antibody with secondary antibody or anti-calreticulin antibody with secondary antibody or anti-HMGB1 antibody with secondary antibody, were added into each group. CLSM images were then captured.

### 2.6. Bilateral Antitumor Study

Balb/c mice were subcutaneously injected with 4T1 cells into the right flank at a density of 5 × 10^6^ and with 4T1 cells into the left flank at a density of 1 × 10^6^ as primary tumors and abscopal tumors. The mice were firstly divided randomly into the following five different groups (n = 5): (1) PBS; (2) RT (4 Gy); (3) BFL; (4) BL + RT; (5) BFL + RT, where the BTS dose was 15 mg/kg. Next, the body weight and tumor volume were measured every third day. Mice were sacrificed at 15 days post-treatment. Main organs and tumors were harvested for further investigation.

Other experimental details are included in the Supporting Information file.

## 3. Results and Discussion

### 3.1. Characterization of BL and BFL

**Figure 1:**
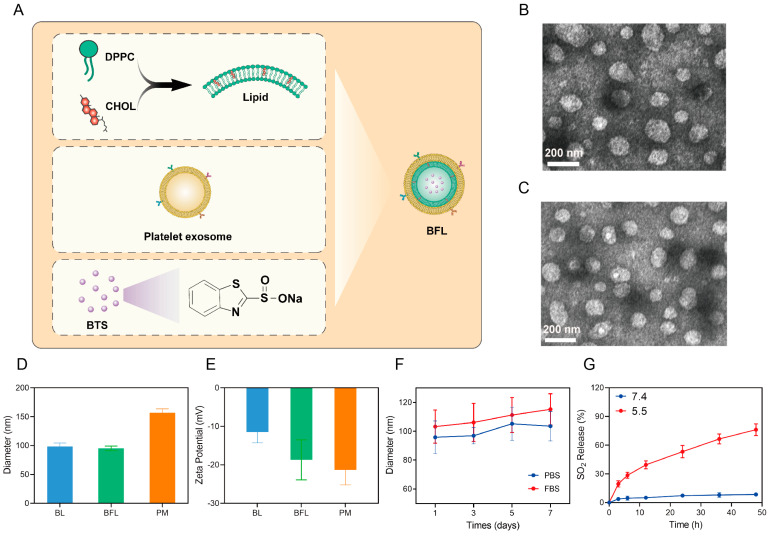
BFL characterization. (**A**) Synthesis procedure of BFL. Representative TEM images of (**B**) BL and (**C**) BFL. (**D**) Average size comparison of BL, BFL, and PM. (**E**) Zeta potential measurements for each sample. (**F**) Monitoring of BFL diameter over time. (**G**) SO_2_ release profile of BFL in pH 5.5 (red) and 7.4 (blue) media.

The synthesis procedure is demonstrated in Figure 1A. Following the encapsulation of BTS in single lipid or fusion liposomes, BL and BFL were synthesized. The morphology of BL and BFL is depicted in Figure 1B,C, respectively. The polydispersity indices are 0.19 ± 0.01 and 0.20 ± 0.02 for BL and BFL, respectively. Both BL and BFL exhibited a consistent spherical structure with a diameter of approximately 100 nm, in contrast to the PM with a diameter of 150 nm (Figure 1D). Previous research reported that a generous average diameter of exosome was found to be approximately 100 nm, consisting with the measured diameter of BL and BFL [40]. The zeta potential of BL measured at −11.5 ± 2.8 mV contrasts with BFL’s zeta potential of −18.7 ± 5.2 mV. The latter aligns more closely with the zeta potential of PM (−21.9 ± 3.9 mV), indicating the successful coating of PM onto BL for the synthesis of BFL (Figure 1E). Additionally, further investigation into the stability of BFL was conducted. The results depicted in Figure 1F demonstrated that BFL exhibited satisfactory stability in both PBS and cell culture medium containing 10% FBS, as evidenced by negligible changes in diameter observed over a 7-day monitoring period. Vascular metabolism of BTS is rapid due to its slow release in water, which limits the treatment efficacy of BTS. The exosome camouflaging can kill two birds with one stone by prolonging circulation of BTS while enhancing tumor targeting efficacy. We evaluated the generation of SO_2_ in solution at different pH values, considering that BTS can produce SO_2_ in acidic conditions. As shown in Figure 1G, a significantly larger amount of SO_2_ was released in an acidic environment with a pH of 5.5, compared to a neutral solution. These results are consistent with previously reported BTS-based nanoplatforms which generate SO_2_ in acidic solutions, reaching a relative intensity of approximately 1.8 in acidic solution compared with that of 1.2 at a pH value of 7.4 [13]. Therefore, we successfully synthesized uniform spherical BFL structures with a diameter of 100 nm, which demonstrated the capability to generate a substantial amount of SO_2_ in acidic solutions. These results provide possibility for further application of BFL in vitro and in vivo.

### 3.2. BFL Enhances RT In Vitro

**Figure 2:**
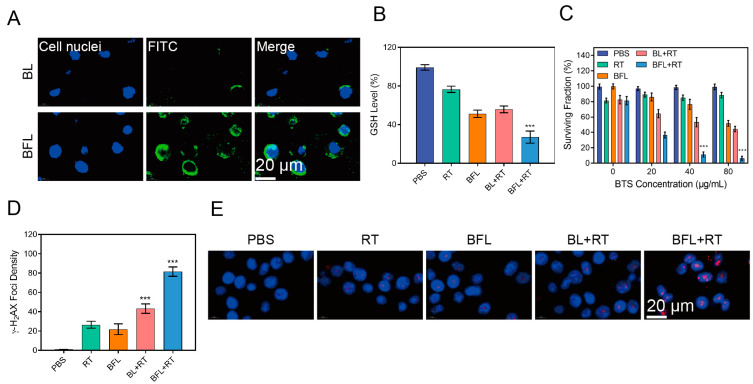

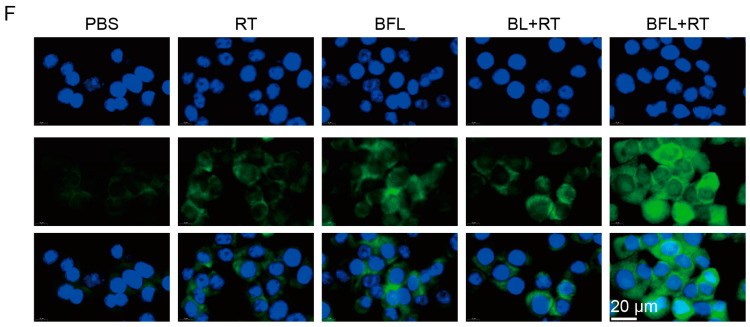
In vitro experiments. (**A**) Fluorescence intensity of 4T1 cells incubated with BL and BFL for 3 h (blue: cell nuclei; green: FITC). (**B**) GSH levels in 4T1 cells under various treatments. (**C**) Survival fractions in all groups with varying BTS concentrations. (**D**) Quantification of γ-H_2_AX foci intensity and (**E**) confocal fluorescence images (Blue: cell nuclei; red: γ-H_2_AX). (**F**) ROS production detected using the DCFH-DA probe (blue: cell nuclei; green: DCFH-DA). Significance between every two groups was calculated by the student’s *t*-test. *** *p* < 0.005.

Following the successful fabrication of BL and BFL, we investigated the radiosensitization effect in vitro. Previous research reported a Au–Ag-BTS-based nanocomplex for SO_2_ gas therapy, which showed a passive targeting ability to tumor cells [41]. Initially, we examined the cellular uptake of FITC-labeled BL and FITC-labeled BFL in 4T1 cells using CLSM. As shown in Figure 2A, following the removal of residue nanoparticles, the green fluorescence intensity, indicative of nanoparticles surrounding cell nuclei, was higher in the BFL group compared to BL. The results reflect higher cellular uptake of active targeting compared with passive targeting. Taking into account the reported ability of SO_2_ to consume GSH [4], the GSH content in each treatment group was evaluated. As shown in Figure 2B, BFL alone led to a reduction in intracellular GSH levels. In contrast, a significant decrease was observed in the BFL + RT group, indicating the apparent GSH consumption ability of BFL + RT. Motivated by these findings, the survival rate of cells was examined across different treatment categories, employing BTS concentrations ranging from 0 to 80 μg/mL and exposing them to an RT. In the RT group, cell viability reached approximately 80%. Noticeably, the addition of BL with RT significantly suppressed tumor cell viability. Moreover, cell viability in the BFL + RT group exhibited remarkable inhibition compared to other groups at BTS concentrations of 40 and 80 μg/mL. Further assessment of double-strand DNA breaks was conducted using γ-H_2_AX staining. 4T1 cells were co-incubated with either BL or BFL, followed by irradiation. The semi-quantification of γ-H_2_AX foci density is shown in Figure 2D. RT and BFL showed slight fluorescence intensity. However, the red fluorescence signal indicating DNA damage was stronger in the BL + RT group compared to the RT group. In contrast, 4T1 cells treated with BFL followed by RT displayed a notable increase in red fluorescence intensity. The representative CLSM images across all treatment groups depicted in Figure 2E were consistent with the semi-quantification results. Subsequently, we evaluated ROS generation using the DCFH-DA probe. As anticipated, cells exposed to RT exhibited moderate ROS generation. However, when cells were pre-treated with BFL before RT, a significant increase in green fluorescence was observed surrounding the cell nuclei, indicating a higher level of ROS production in the BFL + RT group. These results suggest that BFL was endowed with specific tumor targeting ability, enhancing cellular uptake of BFL by 4T1 cells. BFL-enhanced RT also has the potential to consume GSH, which might be attributed to impairing self-repair of tumor cells. Most DNA severe damage and apparent ROS generation was observed in the BFL + RT group, consistent with the effect of significant proliferation inhibition, ultimately contributing to tumor cell death. Cisplatin has been widely applied in clinic chemoradiotherapy against cancer. However, the ROS generation induced by cisplatin under 5 Gy of radiotherapy was relatively weaker than the BFL + RT group [42]. Hereby, the BFL + RT group showed potential in tumor cell elimination in vitro.

### 3.3. BFL Enhances RT to Provoke an Immune Response In Vitro

**Figure 3:**
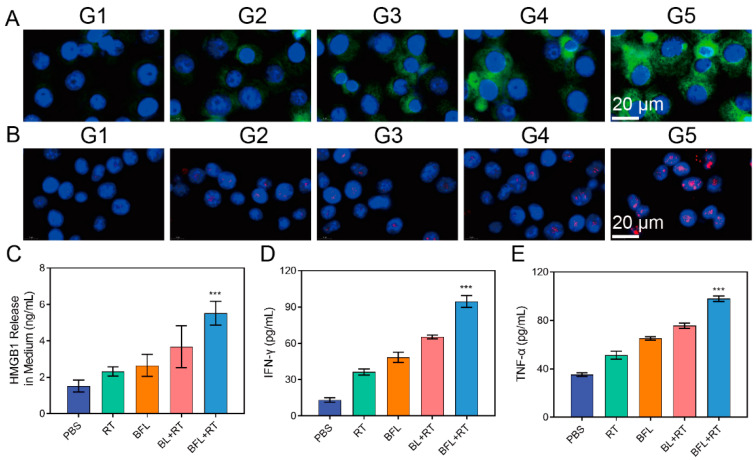
In vitro ICD effect. (**A**) CRT analysis by fluorescence staining (blue: cell nuclei; green: CRT). (**B**) Immunofluorescence analysis of HMGB1 content differences (blue: cell nuclei; red: HMGB1). (**C**) HMGB1 levels in cell supernatant. Secretion of (**D**) IFN-γ and (**E**) TNF-α after various treatments. G1: PBS; G2: RT; G3: BFL; G4: BL + RT; and G5: BFL + RT. Significance between every two groups was calculated by the student’s *t*-test. *** *p* < 0.005.

Motivated by the improved effectiveness of RT by BFL, we proceeded to assess the immune response at the cellular level. ICD can promote the maturation of DC, which plays a key role in regulating both innate and adaptive immunity. To be more specific, danger-associated molecular patterns, including calreticulin (CRT) exposure and high mobility group box 1 (HMGB1), are closely associated with ICD responses. Following different treatments, the levels of CRT and HMGB1 in the medium were assessed, as depicted in Figure 3A,B, respectively. Notably, a high expression of CRT exposure was observed in the BFL + RT group, characterized by a significant increase in green fluorescence intensity on the surface of 4T1 cells. Concurrently, HMGB1 was significantly upregulated in the cell nuclei of the BFL + RT group, a finding consistent with the quantification results presented in Figure 3C. Significantly higher secretion of cytokines, including IFN-γ and TNF-α, in the medium, which promote DC maturation and T cell activation, was also observed in the BFL + RT group (Figure 3D,E). This suggests that outstanding CRT exposure and HMGB1 release was found in the BFL + RT group, which activates antigen presentation and provokes a robust immune response in vitro.

### 3.4. Radiosensitization by BFL In Vivo

**Figure 4:**
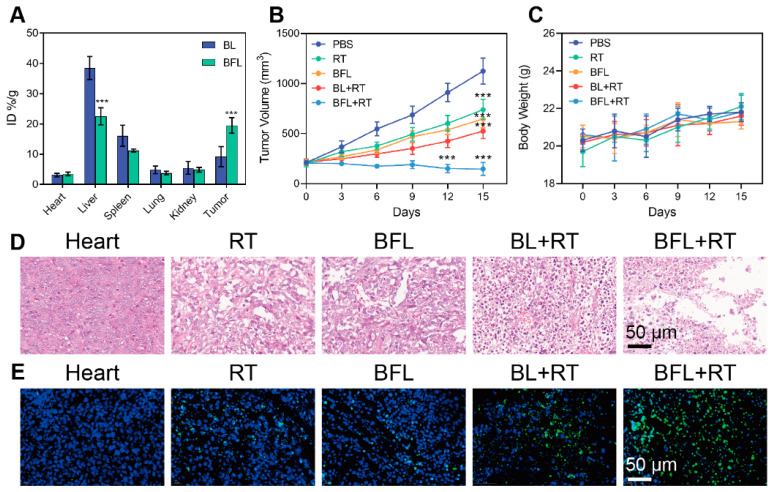
In vivo radiosensitization evaluation. (**A**) Biodistribution following injection of BL or BFL. (**B**) Tumor volume and (**C**) body weight across all groups. (**D**) HE staining and (**E**) TUNEL staining after different treatments (blue: cell nuclei; green: TUNEL). Significance between every two groups was calculated by the student’s *t*-test. *** *p* < 0.005.

Encouraged by the radiosensitization effect observed in vitro, we established a 4T1 tumor-bearing mouse model. Subsequently, we evaluated the tumor-targeting capability of BL and BFL when the tumor volume reached 300 mm^3^. Following the intravenous injection of DiR-labeled BL or BFL for 12 h, tumors were collected for analysis using fluorescence spectrophotometry. As shown in Figure 4A, BL exhibited a tendency for higher accumulation in the lungs compared to BFL, whereas a greater amount of BFL was observed in tumors compared to BL. This suggests a higher tumor-targeting ability of BFL, which can be attributed to the presence of the PM coating. Next, the antitumor efficacy was evaluated in mice subjected to various treatments. It was previously reported that BTS alone demonstrated limited tumor inhibition, reaching about five times the relative tumor volumes at the end of observation [13]. Therefore, the treatment by BTS alone can barely ablate tumors. As shown in Figure 4B, compared with the PBS group, a slight inhibition of tumor growth was observed in the RT group. A similar tumor suppression effect was observed in the BL group. In contrast, the BFL + RT group demonstrated excellent antitumor efficacy. Biosafety should be taken into consideration when assessing the therapeutic efficacy. Another SO_2_ prodrug, PDDN, showed little system toxicity since little body weight loss was observed [43]. Additionally, there was no apparent difference in body weight among the groups, indicating negligible systematic toxicity (Figure 4C). Further investigation from the HE staining assay depicted in Figure 4D showed a crinkled, deformed, and even dissolved morphology of tumor cells after treatment with BFL + RT. Additionally, TUNEL-positive staining was more significant in the BFL + RT group, indicating severe apoptosis and necrosis (Figure 4E). These results imply that BFL demonstrated significant tumor targeting ability due to mimicking disguise by exosome. The strategy by BFL enhanced RT showed excellent tumor ablation effect by causing severe tumor cell damage without significant toxicity.

### 3.5. In Vivo Antitumor Efficacy Evaluation

**Figure 5:**
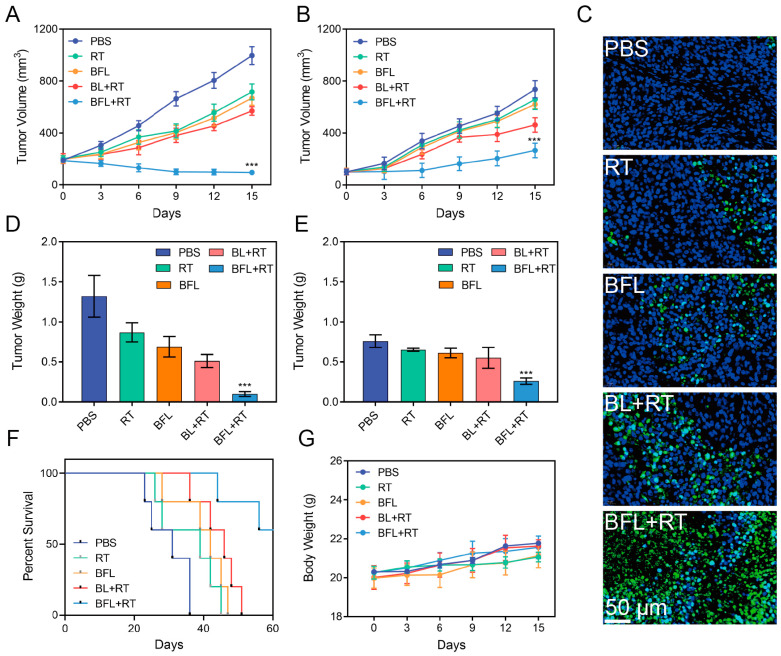
In vivo antitumor effect. (**A**) Primary tumor and (**B**) distant tumor volume curves in bilateral 4T1 tumor-bearing mice post various treatments. (**C**) CLSM images obtained by the TUNEL technique (blue: cell nuclei; green: TUNEL). (**D**) Primary tumor and (**E**) distant tumor weights after treatments. (**F**) Survival rate and (**G**) body weight across all treatment groups. Statistical significance was calculated via one-way ANOVA with Tukey’s test: *** *p* < 0.001.

Subsequently, a 4T1 bilateral tumor model was established in BALB/c mice to confirm the antitumor efficacy. Changes in tumor volume and body weight were monitored every third day. In the PBS group, both primary and distant tumors grew out of control, while the RT or BFL group was able to suppress tumor growth to some extent. However, they could not alleviate the rapid progression of tumors in the distant tumor site (Figure 5A,B). Similarly, the BL + RT group exhibited significant tumor suppression in both primary and distant tumors, confirming the moderate immune response provoked by BL + RT. In contrast, the BFL + RT group elicited significant antitumor efficacy, resulting in a remarkable reduction of both primary and distant tumors. TUNEL staining was performed on primary tumors collected from each group. Moderate cell apoptosis was observed in the RT or BFL group. However, a more pronounced green fluorescence intensity, representing positive TUNEL staining, is shown in Figure 5C. Nevertheless, the highest number of apoptotic cancer cells was observed in the BFL + RT group. Consistent with the volumes of primary and distant tumors obtained in various groups, the weights of the collected tumors are illustrated in Figure 5D,E. During the 60-day observation period, while mice in other groups were sacrificed, 60% of the mice survived due to tumor alleviation, as shown in Figure 5F. At the same time, there were no noticeable significant changes observed among all treatment groups, as depicted in Figure 5G. Therefore, the presence of BFL significantly contributed to the therapeutic effect of RT. Meanwhile, treatment of BFL enhanced RT displayed abscopal effect by suppressing distant tumor growth. However, further investigation is urgent for the mechanism of this abscopal effect.

### 3.6. Immune Response Activated by BFL + RT Contributes to the Inhibition of Tumor Metastasis

**Figure 6:**
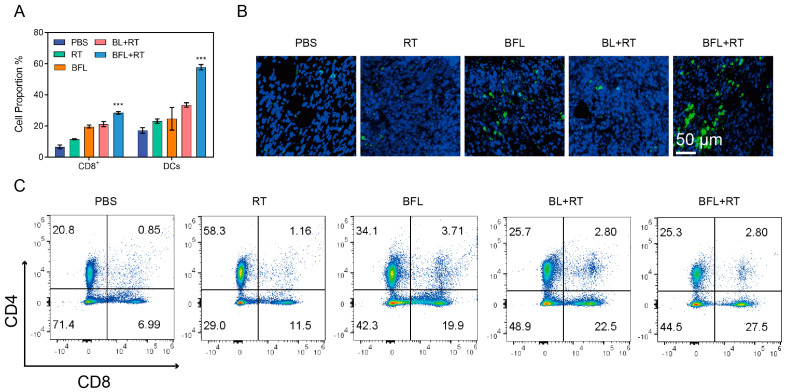
Immune response of BFL-nhanced RT. (**A**) Statistical analysis of DC in lymph nodes and CD8^+^ T cells in distant tumor tissues. (**B**) Immunofluorescence of CD8^+^ T cells across all groups. (blue: cell nuclei; green: Anti-CD8 antibody). (**C**) Proportion of CD8^+^ T cells and CD 4^+^ T cells in distant tumor tissues. Statistical significance was calculated via one-way ANOVA with Tukey’s test: *** *p* < 0.001.

Tumor metastasis may occur post-treatment, posing a threat to prognosis in clinical settings. Tumor tissues and lymph nodes were harvested from each group for analysis. Subsequently, we assessed the population of CD80^+^CD86^+^ in the lymph nodes. As crucial antigen-presenting cells, DC play a significant role in antigen processing to stimulate T cells. As shown in Figure 6A, the BFL + RT group exhibited notably enhanced DC maturation compared to other groups, consequently promoting the activation of CD8^+^ T cells. The expression of CD8^+^ staining was slightly enhanced in mice that received BL + RT treatment (Figure 6B). At the same time, the highest intensity of green fluorescence, representing CD8^+^ staining, was observed in distant tumor slices from the BFL + RT group. The evaluation of CD8^+^ cytotoxic T lymphocytes was conducted through flow cytometry analysis. A representative flow cytometry pattern is demonstrated in Figure 6C. Remarkably, the BFL + RT group exhibited significant potential in promoting T cell activation, consistent with the quantification results presented in Figure 6C. The percentage of tumor-infiltrating CD8^+^ T cells in distant tumors notably increased after treatment with BFL + RT, reaching a population of 30% in the BFL + RT group. Hereby, the DC maturation of BFL promoted CD8^+^ T activation in distant tumors, which leads to tumor suppression. It is worth mentioning that although chemoradiotherapy by cisplatin demonstrated remote tumor suppression, the elevation of local immune cell infiltration is restrained [44]. The results validate our strategy of employing BFL + RT to evoke a systemic immune response through DC maturation and T cell activation, hereby leading to obvious tumor suppression and metastasis inhibition.

### 3.7. Biosafety Test

In addition to the minimal fluctuation observed in body weight among the treatment groups, indicating no significant systemic toxicity, we proceeded to evaluate the biosafety of BFL in healthy mice. Blood hematology and biochemistry analyses, along with HE staining of major organs, were conducted, which revealed no significant differences between the BFL group and the control group (Figures S1 and S2). These findings indicate the safety of intravenous administration of BFL.

## 4. Conclusions

A customized nanocarrier, BFL, incorporating liposome/PM camouflage for delivering the SO_2_ prodrug BTS, was synthesized for the suppression of 4T1 tumors and activation of immunogenic responses alongside RT. The BTS-loaded nanodrug was demonstrated to have a uniform diameter, excellent stability, and satisfactory biocompatibility, exhibiting remarkable tumor-specific targeting ability and enhancing cellular uptake. The mechanism of radiosensitization of BFL was investigated. BFL targets the tumor site and enters tumor cells, facilitating the release of BTS. Functioning as a prodrug of SO_2_, BTS within BFL generates SO_2_ in an acidic environment. This process leads to the depletion of GSH, thereby preventing DNA self-repair induced by RT. The enhancement of RT by BFL has a significant impact on alleviating the immunosuppressive TME, resulting in DC maturation and T cell infiltration as well as secretion of immune response-related cytokines. In particular, BFL + RT demonstrated remarkable antitumor efficacy and effectively inhibited the growth of remote tumors. The combination of BFL and RT showed potential in activating immunogenic responses, making it promising for both tumor suppression and inhibition of metastasis.

## Data Availability

The data presented in this study are available on request from the corresponding author.

## Supplementary Materials

The following supporting information can be downloaded at: https://www.mdpi.com/article/10.3390/pharmaceutics16060833/s1, Figure S1: HE staining of main organs collected from mice injected with PBS or BFL; Figure S2: Blood hematology and biochemistry analyses of blood collected from mice injected with PBS or BFL.
