# Supplementary material for: Personalized SO2 Prodrug for pH-Triggered Gas Enhancement in Anti-Tumor Radio-Immunotherapy
Source: Pharmaceutics. 2024 Jun 19;16(6):833. doi: 10.3390/pharmaceutics16060833 (PMC11207922; doi:10.3390/pharmaceutics16060833)
Supplement: Supplementary file 1 [file pharmaceutics-16-00833-s001.zip › pharmaceutics-3019602-supplementary.pdf]

Personalized SO_2 prodrug for Ph-triggered gas enhancement in anti-tumor radio-immunotherapy

Experimental Procedures

Materials and reagents.

Cholesterol (Chol) and 1,2-dipalmitoyl-sn-glycero-3-phosphocholine (DPPC) were purchased from A.V.T (Shanghai, China). The HMGB1 ELISA kit was purchased from Beijing Solarbio Science & Technology Co.,Ltd. (China). All of the aqueous solutions were prepared using purified deionized (DI) water purified with a purification system (Direct-Q3, Millipore, USA). The other solvents used in this work were purchased from Sinopharm Chemical Reagent (China) and Aladdin-Reagent (China).

Cell culture

4T1 cell line were obtained from the Cell Bank of the Chinese Academy of Sciences and incubated in RPMI-1640 medium supplemented with 10% FBS in a humidified atmosphere.

Detection of Intracellular GSH

The commercially available GSH assay kit was used to detect the depletion of GSH. 4T1 CSC (8×10^4 per plate) were incubated with Five different group: (1) PBS; (2) RT (4Gy); (3) BFL; (4) BL+RT; (5) BFL +RT. The BTS concentration was 50 μg/mL. The RT was conducted 2h after different treatment. Then, the cells were suspended in 1 mL of PBS and processed by an ultrasound cell crusher. After that, 0.5 mL of the above cells was added to 2 mL of regent one in an assay kit and centrifuged at 3500 rpm for 10 min. The depletion of intracellular GSH was measured by GSH Content Assay Kit (Solarbio life sciences).

Clonogenic survival assay

In the radiation therapy experiment, 500 cells were seeded in eight 6-wells plates incubating for 24 h, then the cells were incubated with Five different group: (1) PBS; (2) RT (4Gy); (3) BFL; (4) BL+RT; (5) BFL +RT. The RT was conducted 2h after different treatment. To allow formation of colonies, after radiation, the cells were then incubated for another 10 days, without changing the media. To determine the clonogenic survival rate, cultures were first fixed with paraformaldehyde, and then stained with trypan blue. Colonies with greater than 50 cells were counted under

the microscope, and the survival fractions (SF) were calculated using the formula SF = colonies counted/cells seeded.

Animal tumor models

Female Balb/c mice aged 4-5 week were purchased from Vital River Company (Beijing, China). 100 μL of 4T1 cell suspension (5×10^6 cells) were subcutaneous injected into each mouse to establish the tumor models. The animal experiments were carried out according to the protocol approved by the Ministry of Health in People's Republic of PR China.

***In vivo* biodistribution study**

The 4T1 tumor model was used. When tumors reached 300mm^3, tumor bearing mice (n = 3) received an intravenous (i.v.) of 100 μL PBS containing DiR labeled BL or BFL (with a DiR dose of 5mg/kg). Mice were sacrificed at 12 h after injection to collect the tumors and major organs for fluorescence spectrophotometry analysis and expressed as DiR weight in per gram of tissue.

Tumor tissue immunofluorescence staining

Tumor tissues were weighed, and fixed in 4% neutral buffered formalin, processed routinely into paraffin, and sectioned at 4 μm. Then the primary tumor sections were stained with TUNEL and finally examined by using fluorescence microscope (IX81, Olympus, Japan). The distant tumor sections were stained with anti-CD8 Antibody and finally examined by using fluorescence microscope (IX81, Olympus, Japan).

***In vivo* immune response after different treatments**

To examine DC maturation in vivo, the spleens were harvested. The frequency of DC maturation was then examined by CD11c+ cell sorting kit (NovoBiotechnology Co., Ltd.) and flow cytometry after immunofluorescence staining with anti-CD80-BV421(BD Bioscience) and anti-CD86-APC (ab218757) antibodies**.** To detect T cell content, distant tumors were harvested from mice in different groups and treated with Mouse CD3$^+$T cell sorting kit (Suzhou Beaver Biomedical Engineering Co., Ltd), anti-CD8-Alexa Fluor 488 (BD Biosciences, 557704) and anti-CD4-PE (ab252151) according to the manufacturer's protocols. To analysis treatment-induced cytokine secretion, whole blood was collected from mice at 21 days post treatment. The serum concentration of proinflammatory cytokines including TNF-α and IFN-γ were then analyzed with ELISA kits (Neobioscience Co., Ltd., China) according to the manufacturer's instructions.

Statistical analysis

Data analyses were conducted using the GraphPad Prism 5.0 software. Significance between every two groups was calculated by the student's t-test. *P < 0.05, **P < 0.01, ***P < 0.005.

Supplementary figures

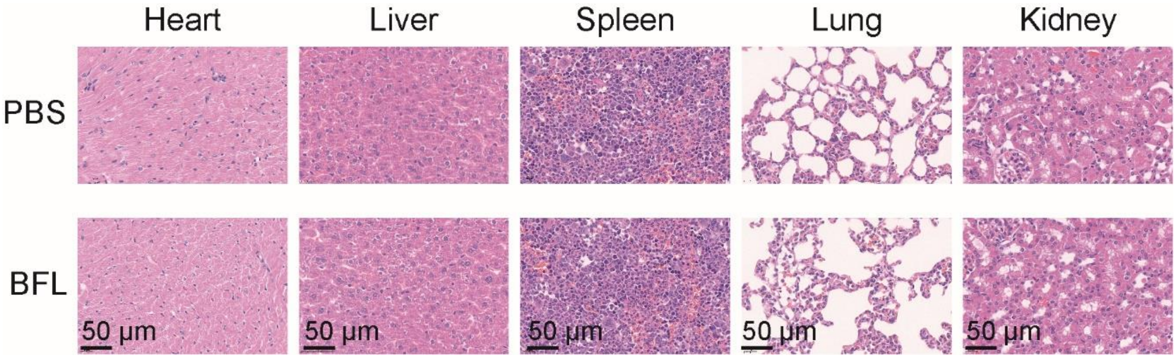

Figure S1. HE staining of main organs collected from mice injected with PBS or BFL.

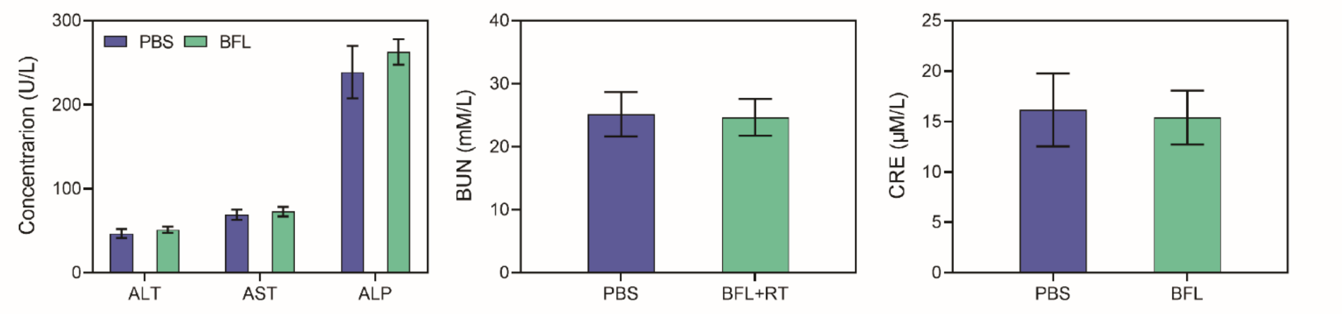

Figure S2. Blood hematology and biochemistry analyses of blood collected from mice injected with PBS or BFL.
